# Fast-evolving noncoding sequences in the human genome

**DOI:** 10.1186/gb-2007-8-6-r118

**Published:** 2007-06-19

**Authors:** Christine P Bird, Barbara E Stranger, Maureen Liu, Daryl J Thomas, Catherine E Ingle, Claude Beazley, Webb Miller, Matthew E Hurles, Emmanouil T Dermitzakis

**Affiliations:** 1 The Wellcome Trust Sanger Institute, Wellcome Trust Genome Campus, Hinxton, CB10 1SA, UK; 2 Center for Biomolecular Science and Engineering, University of California Santa Cruz, Santa Cruz, CA 95064, USA; 3 Department of Computer Science & Engineering, Pennsylvania State University, University Park, PA 16802, USA

## Abstract

**Background:**

Gene regulation is considered one of the driving forces of evolution. Although protein-coding DNA sequences and RNA genes have been subject to recent evolutionary events in the human lineage, it has been hypothesized that the large phenotypic divergence between humans and chimpanzees has been driven mainly by changes in gene regulation rather than altered protein-coding gene sequences. Comparative analysis of vertebrate genomes has revealed an abundance of evolutionarily conserved but noncoding sequences. These conserved noncoding (CNC) sequences may well harbor critical regulatory variants that have driven recent human evolution.

**Results:**

Here we identify 1,356 CNC sequences that appear to have undergone dramatic human-specific changes in selective pressures, at least 15% of which have substitution rates significantly above that expected under neutrality. The 1,356 'accelerated CNC' (ANC) sequences are enriched in recent segmental duplications, suggesting a recent change in selective constraint following duplication. In addition, single nucleotide polymorphisms within ANC sequences have a significant excess of high frequency derived alleles and high F_ST_values relative to controls, indicating that acceleration and positive selection are recent in human populations. Finally, a significant number of single nucleotide polymorphisms within ANC sequences are associated with changes in gene expression. The probability of variation in an ANC sequence being associated with a gene expression phenotype is fivefold higher than variation in a control CNC sequence.

**Conclusion:**

Our analysis suggests that ANC sequences have until very recently played a role in human evolution, potentially through lineage-specific changes in gene regulation.

## Abstract 2

Over 1,300 conserved non-coding sequences were identified that appear to have undergone dramatic human-specific changes in selective pressures; these are enriched in recent segmental duplications, suggesting a recent change in selective constraint following duplication.

## Background

The manner in which the expression of genes is regulated defines and determines many of the cellular and developmental processes in an organism. It has been hypothesized that variation in gene regulation is responsible for much of the phenotypic diversity within and between species [1]. In particular, it was proposed a few decades ago that the phenotypic divergence between human and chimpanzees is largely due to changes in gene regulation rather than changes in the protein-coding sequences of genes [2]. Although it has been long recognized that regulatory sequences play an important role in genome function, the fine structure and evolutionary patterns of such sequences are not well understood [3], mainly because such sequences have a much more complex functional code and appear not to be restricted to particular sequence motifs. One of the most powerful approaches with which to identify regulatory sequences has been to use multiple species comparative sequence analysis to look for conserved noncoding (CNC) sequences [4], but these sequences represent only a subset of regulatory elements in the genome and only a subset of them are regulatory elements [5].

CNC sequences are distributed throughout the genome in a manner independent of gene density [6,7]. Studies of nucleotide variation have revealed strong selective constraints on CNC sequences in human populations [8], and so there is little doubt that a large number of them have a functional role. The abundance and genomic distribution of CNC sequences has raised intriguing questions about the functions of such sequences in the genome. Although a small fraction of the CNC sequences can be associated with transcriptional regulation (most of the most highly conserved examples of CNC sequences appear to be enhancers of early development genes [5,9]), there remains a large number of CNC sequences with unexplained function.

Although the identification of CNC sequences relies on sequence conservation, it is conceivable that some of the most interesting functional noncoding elements are also evolving under positive (directional) selection in particular lineages. Studies in *Drosophila *have suggested that such a pattern exists in untranslated regions and in some introns and intergenic DNA [10]. Moreover, loss-of-function mutations as well as mutations that lead to gain of novel functions are also likely to contribute to evolutionary change [11,12]. A relatively recent model for the evolution of novel gene function following gene duplication proposes that the reciprocal degeneration of regulatory elements after duplication (duplication-degeneration-complementation) [13] could drive gene subfunctionalization, and an older model of gene duplication proposed an important role for positive selection after duplication [14-16]. All of the above evolutionary processes could contribute to phenotypic evolution in the human lineage, and would result in a lineage-specific acceleration of the substitution rate of associated functional noncoding DNA.

In the present study we conducted an analysis of lineage-specific acceleration of previously identified CNC sequences in vertebrates. By comparing the CNC sequences of three genomes - human, chimpanzee and macaque - we identify 1,356 CNC sequences that have an excess of human-specific substitutions relative to the chimpanzee lineage. By analyzing the genomic distribution and nucleotide variation of these fast-evolving (accelerated) CNC sequences, we find that significant numbers of them are found in the most recent (mostly human-specific) segmental duplications, and single nucleotide polymorphisms (SNPs) within them are associated with changes in gene expression. We also find a strong signal of recent directional selection in the human lineage.

## Results

### Searching for fast-evolving (accelerated) conserved noncoding sequences

**Figure 1:**
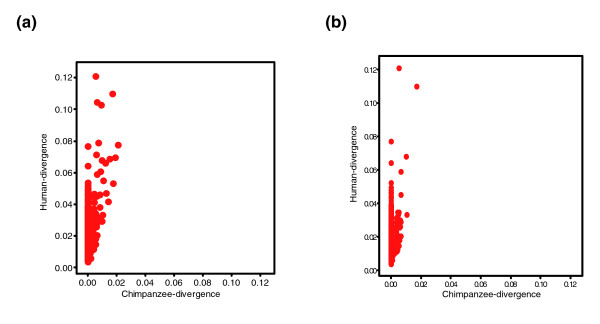
Substitution rates of 1,356 human-specific ANC sequences. Shown are the relative rates (*P *distance) of substitutions of **(a) **the 1,356 accelerated noncoding (ANC) sequences in the human (y-axis) and chimpanzee (x-axis) lineages, and **(b) **the 1,145 ANC sequences excluding those within potential confounding features (segmental duplications, copy number variants, pseudogenes, and retroposons).

We have selected 304,291 of the most conserved noncoding sequences of at least 100 base pairs (bp) in length to look for evidence of accelerated substitution rate in the human lineage (see Materials and methods, below), by comparing the orthologous sequences of CNC sequences between human and chimpanzee. We used a χ^2^-based test to detect regions of CNC sequence that are diverging at an accelerated rate in either the human or chimpanzee lineage [17]. The test requires at least four substitutions between human and chimpanzee. Of the 304,291 CNC sequences, only 26,475 have at least four human-chimpanzee substitutions. For those 26,475 CNC sequences, we generated human-chimpanzee-macaque three-way alignments to infer the direction of substitutions, and performed Tajima's one-tailed χ^2 ^test to detect human-specific or chimpanzee-specific substitution rate acceleration, applying the Yate's correction for continuity to correct for small substitution counts [17]. The chosen *P *value threshold was *P *= 0.08, because it was the *P *value with the minimum false discovery rate (FDR; see Materials and methods, below) in the range of *P *values between 0.05 and 0.15 (FDR = 75%). At this threshold we detected a total of 2,794 (10.6%) accelerated CNC sequences (hereafter referred to as accelerated noncoding [ANC] sequences) in either the human (1,356 ANC sequences [5.1%]) or the chimpanzee (1,438 ANC sequences [5.3%]) lineage (Figure 1a) with *P *≤ 0.08, whereas we expected only 2,118 in total by chance. The FDR of 75% is likely to be an overestimate because the Yate's correction is generally considered conservative.

Comparison of the human and chimpanzee chromosomes in the alignments reveals that only 20 out of 1,356 are not on the expected syntenic chromosome (Additional data file 1). We also conducted visual and manual examination of a random sample of 5% of the ANC sequences across the whole spectrum of significance (Additional data file 1) to confirm that the signals we detect are not a result of misalignments, and we have concluded that this is very rare (only two out of 72 cases are potentially problematic). Some of the ANC sequences overlap with features that could potentially create such patterns (segmental duplications, retroposed genes, and pseudogenes), but in all of the cases that we tested the result cannot be explained by misalignment. In fact, if we exclude sequences that could generate potential alignment artefacts (segmental duplications, retroposed genes, and pseudogenes [see below]), we then detect 1,145 human ANC sequences (Figure 1b) relative to 18,289 power CNC sequences. The FDR is estimated at 40% (P < 0.05), which suggests that 688 (60%) of ANC sequences are true positives, which is a larger proportion than estimated above. We discuss below the relevance of such overlaps to real biological signals and hence their inclusion. However, we also perform all of the analysis (see below) excluding the ANC sequences in the above features to confirm the validity of the obtained results.

**Figure 2:**
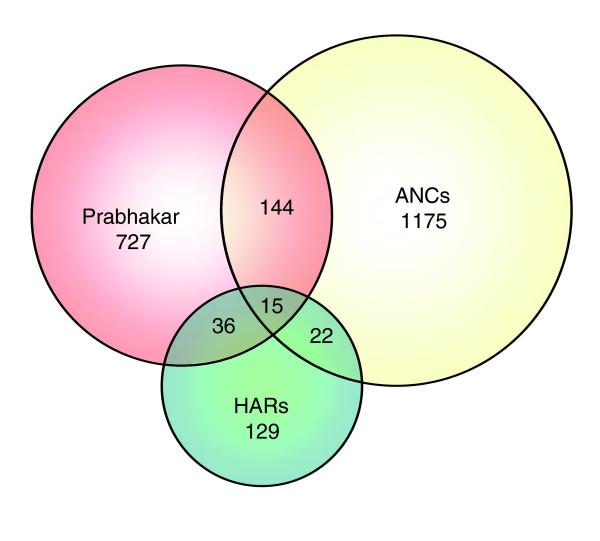
Venn diagram of overlap between accelerated sequences in the three studies. The figure shows the overlap between the present study (yellow), the study by Pollard and coworkers [18] (green), and the study by Prabhakar and colleagues [19] (pink). ANC, accelerated noncoding; HAR, human accelerated region.

Two recent studies [18,19] have also described ANC sequences in the human genome. A total of 37 of the 202 human accelerated regions (HARs; 18%) in the Pollard study [18] and 159 of the 992 accelerated conserved noncoding sequences (CNSs; 16%) in the Prabhakar study [19] overlap our set of ANC sequences. The overlap between these sets is also low; 51 of the 202 HARs (25%) in the Pollard study [18] overlap the CNSs in the Prabhakar study [19]. The overlap between studies (Figure 2) is highly significant, and all three studies are capturing similar signals but clearly the overlap is incomplete. One explanation for the limited overlap between the three studies is that there are many ANC sequences, most of which cannot be detected because of a lack of power. However, it is difficult to distinguish this possibility from the differences expected as a result of use of three methods that rely on different assumptions. In particular, our study uses a methodology that specifically detects human lineage-specific acceleration relative to the chimpanzee, and the identification of ANC sequences is mutually exclusive in the two species, which is not the case in the two other studies.

Throughout this analysis we use the following sets of DNA sequences as genomic controls, against which we compare the human ANC sequences: the 23,681 nonaccelerated CNC sequences with at least four substitutions sufficient to detect significant acceleration (excluding human and chimpanzee ANC sequences, hereafter referred to as 'power CNC sequences'); and all remaining 277,814 nonaccelerated CNC sequences (excluding power CNC sequences).

### Positive selection versus loss of constraint

The analysis above allows us to identify CNC sequences that have accelerated rates of substitutions in humans relative to chimpanzees. This acceleration can be due either to loss of selective constraint or to positive selection, and the biological interpretation of the two is different. Loss of selective constraint should result in sequences adopting the neutral rate of evolution, whereas sequences under positive selection might be expected to be evolving more rapidly than under neutral evolution. In order to obtain a minimum estimate of the fraction of the 1,356 ANC sequences that are undergoing positive selection, we compared the human lineage-specific substitution rate of ANC sequences with that of 50,846 and 50,627 regions of the same size distribution as the CNC sequences that are 10 kilobases (kb) away and 500 kb from a CNC sequence, respectively, and with at least four substitutions between human and chimpanzee. As a threshold to determine whether an ANC sequence has a substitution rate higher than neutral, we defined the 5% tail of the distributions of human lineage-specific divergence of the two sets. These thresholds are d_0.05 at 10 kb _= 0.0267 and d_0.05 at 500 kb _= 0.0268. A total of 260 (19%) and 259 (19%) ANC sequences have rates higher than these thresholds, respectively, whereas only 5% (68 ANC sequences) are expected by chance. This suggests that at least 191 ANC sequences have undergone sequence divergence consistent with positive selection. If we exclude potentially confounding ANC sequences, then we observe that 200 of the 1,145 ANC sequences (17.5%) have a human lineage-specific rate above the neutral threshold and that this accounts for at least 143 ANC sequences presumably under positive selection.

In an alternative approach, we compared the human lineage-specific rate with the synonymous substitution rate estimated from human and chimpanzee [20], which in some cases may serve as a neutral proxy. The average synonymous substitution rate was computed as Ks = 0.0141 ± 0.0132 (mean ± standard deviation [SD]), and an estimate of the expected human Ks rate is taken as half that. We consider two upper bounds of neutral rate as Ks_2 SD _= mean + 2 SD = 0.0203 and Ks_3 SD _= mean + 3 SD = 0.0270. With Ks_2 SD _and Ks_3 SD_, 515 ANC sequences (38%) and 253 ANC sequences (18%), respectively, are estimated to have undergone positive selection. Similar results are obtained if we consider the observed distribution of Ks values to determine the 95% (*P *< 0.05) and 99% (*P *< 0.01) upper confidence limits. We conclude that at least 15% and potentially more than one-third of the ANC sequences are evolving faster than the neutral substitution rate. Synonymous sites can be constrained but the fact that all three methods give similar results suggests that 15% to 19% of ANC sequences have substitutions rates above what is expected by neutral evolution.

### Genomic location of accelerated noncoding sequences

We investigated the possibility that ANC sequences are degenerate regulatory elements associated with subfunctionalized genes or elements that have decayed in function following duplication in a manner similar to pseudogenes. We explored the distribution of ANC sequences, power CNC sequences, and nonaccelerated CNC sequences in recent segmental duplications of the human genome, as defined in recent studies [21,22]. Approximately 5% to 6% of the genome is included in segmental duplications, but we find 8% of the ANC sequences, 10% of the power CNC sequences, and only 5% of nonaccelerated CNC sequences (Table 1) within segmental duplications. This suggests an enrichment of ANC sequences and power CNC sequences in segmental duplications, and this is significantly different from the density of nonaccelerated CNC sequences in segmental duplications (χ^2 ^test, *P *< 10^-4^).

**Table 1:** Percentage overlap between sets of genomic features with ANC sequences, power CNC sequences, and nonaccelerated CNC sequences

Sequence	All	Segmental duplication	CNV	Segmental duplication or CNV	Pseudogene	Retroposed gene	Pseudogene or retroposed gene	Segmental duplication, CNV, pseudogene, or retroposed gene
ANC	1,356	108 (8%)	62 (5%)	138 (10%)	72 (5%)	102 (8%)	111 (8%)	211 (16%)
Power CNC	23,681	2,346 (10%)	1,240 (5%)	3,087 (13%)	2,207 (9%)	3,489 (15%)	3,576 (15%)	5,392 (23%)
Nonacc CNC	277,814	13,889 (5%)	10,514 (4%)	21,874 (8%)	9,094 (3%)	15,988 (6%)	16,836 (6%)	32,405 (12%)

ANC, accelerated noncoding; CNC, conserved noncoding; CNV, copy number variant.

**Figure 3:**
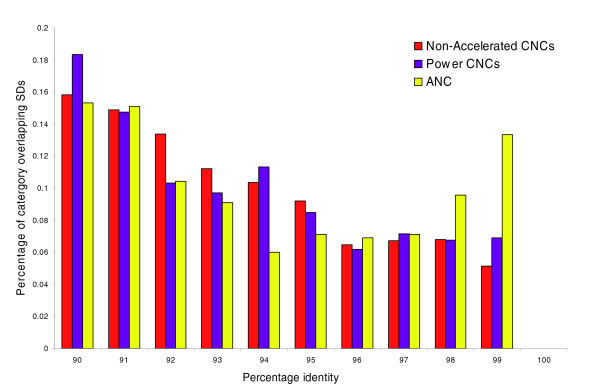
Segmental duplication divergence in ANC and CNC sequences. The figure shows that the divergence of paralogs in segmental duplications (SDs) where conserved noncoding (CNC) sequences (red) and power CNC sequences (purple) are found is skewed to high divergence values, whereas the accelerated noncoding (ANC) sequences (yellow) have a strong enrichment in recent segmental duplications, as expected if the acceleration is due to a recent change in selective forces (positive selection or loss of selective constraint).

We subsequently considered the age of the segmental duplications containing ANC sequences, power CNC sequences, and nonaccelerated CNC sequences, by comparing the distribution of percentage identity between paralogs of segmental duplications overlapping each of the three sets above. The distribution for segmental duplications containing ANC sequences reveals that ANC sequences are highly enriched within recent segmental duplications of low divergence (<2%; Figure 3). The distributions of the two controls are both significantly skewed toward an excess of old and highly diverged segmental duplications (Mann-Whitney U-test; *P *< 10^-4^). This strongly suggests that some ANC sequences have undergone modification of their selective pressures (either loss of selective constraint or positive selection) after very recent duplication.

To test for enrichment of ANC sequences in variable genomic duplications segregating in human populations, we intersected ANC sequences, power CNC sequences, and nonaccelerated CNC sequences with human copy number variants (CNVs) from a public database (Database of Genomic Variants in Toronto [23]). The enrichment we observed was entirely due to high overlap between CNVs and segmental duplications, suggesting no enrichment of ANC sequences in CNVs *per se*.

We further explored the overlap of ANC sequences, power CNC sequences, and the nonaccelerated CNC sequences with retroposed genes and pseudogenes. Only 8% of ANC sequences overlap these elements, as compared with an overlap of 15% for the power CNC sequences (χ^2 ^test, *P *< 10^-4^; Table 1). This supports the concept that the detection of acceleration in ANC sequences is not due to misalignments, because one of our control sets - the power CNC sequences - are more enriched for retroposed genes and pseudogenes. Normally, most studies exclude such sequences from the analysis because they are considered noise, but in light of recent studies that associated function with repetitive elements [24,25], we retained all ANC sequences and CNC sequences overlapping such elements for subsequent analysis. However, in most cases we also perform the analysis without them to control for any biases that they might introduce.

### Historical and recent patterns of nucleotide variation

We further explored the patterns and levels of nucleotide variation in ANC sequences in human populations to determine whether the processes that shape the evolution of ANC sequences are historical (predating human coalescent time) or recent in human populations. We used the derived allele frequency (DAF) spectrum of SNPs from the phase II HapMap [26,27]. The state of the allele (either derived or ancestral) was inferred by aligning the SNP position to the chimpanzee genome and using parsimonious assumptions (see Materials and methods, below). Regions with an excess of SNPs with high DAF relative to the expectations of a neutral equilibrium model are likely to be evolving under positive selection [28].

**Figure 4:**
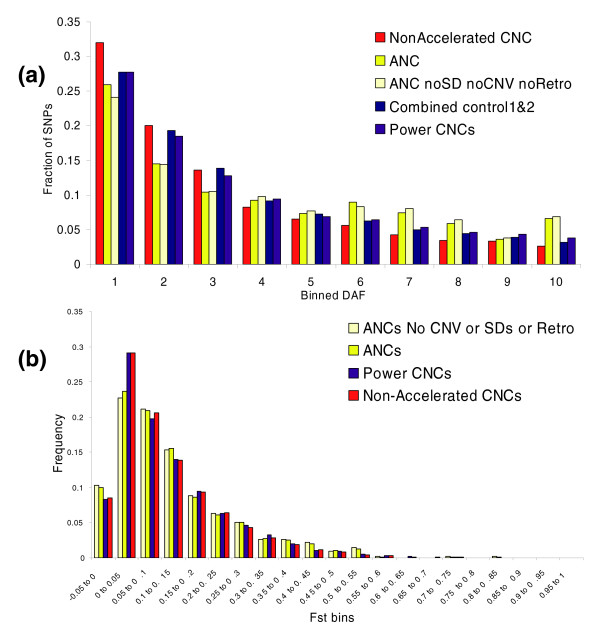
Patterns and levels of nucleotide variation in ANC sequences. **(a) **The comparative derived allele frequency (DAF) spectrums for phase II HapMap single nucleotide polymorphisms (SNPs) in nonaccelerated conserved noncoding (CNC) sequences (*n *= 48,811), accelerated noncoding (ANC) sequences (*n *= 682), ANC sequences outside of segmental duplications, copy number variants (CNVs), retroposed genes or pseudogenes (*n *= 610), in the two controls (*n *= 28,408 and *n *= 28,722), in the power CNC sequences (*n *= 10,882), and in the 60 individuals of the Yoruban (YRI) population. **(b) **The comparative distributions of F_ST _values for all phase II HapMap SNPs in ANC sequences (*n *= 688), ANC sequences outside of segmental duplications, CNVs, retroposed genes or pseudogenes (*n *= 620), power CNC sequences (*n *= 11,267), and nonaccelerated CNC sequences (*n *= 52,210).

We defined five sets of SNPs from the Yoruba (YRI) population of the HapMap [26] project: SNPs within ANC sequences (*n *= 682), power CNC sequences (*n *= 28,722), nonaccelerated CNC sequences (*n *= 48,811), and two new control sets of SNPs (*n *= 28,408 and 28,722) from 1,356 20-kb windows located 500 kb 5' and 3' of the ANC sequences. The DAF spectrum of the ANC sequences has a significant excess of high-frequency derived alleles relative to the DAF spectrum of all control sets (Mann-Whitney U-test, *P *< 10^-4^; Figure 4a). The DAF spectrum of the power CNC sequences is more similar to the neutral controls than to that of the nonaccelerated CNC sequences, possibly suggesting that power CNC sequences are a mix of ANC sequences and nonaccelerated CNC sequences. The other HapMap populations exhibit very similar patterns (data not shown).

Because SNPs in segmental duplications and CNVs can exhibit odd patterns of variation, such as those caused by genotyping errors, we have also performed the analysis excluding any SNPs in ANC sequences that map to segmental duplications, CNVs, or pseudogenes of retroposed genes (*n *= 610), and we observed that the pattern of excess of high-frequency derived alleles remains strong and significant (Figure 4a). This overall analysis suggests that recent, possibly positive selection in ANC sequences has shaped the pattern of nucleotide variation in ways similar to the pattern of fixed nucleotide changes between species.

We then compared the DAF spectrum of SNPs in ANC sequences with those of SNPs within HARs [18] (*n *= 84) and accelerated CNSs [19] (*n *= 328). We observe that SNPs in HARs exhibit an excess of high derived allele frequency, similar to SNPs in ANC sequences, which is consistent with recent positive selection, whereas SNPs in the accelerated CNSs of Prabhakar and coworkers [19] exhibit a pattern more similar to those neutrally evolving (Additional data file 2), indicating once again the heterogeneity of these three sets of accelerated sequences.

### Population differentiation of single nucleotide polymorphisms within accelerated noncoding sequences

In order to further characterize the recent evolutionary pressures on ANC sequences and to detect recent population-specific patterns of selection, we calculated F_ST_, which is a common measure of population differentiation [29], for SNPs in ANC sequences and nonaccelerated CNC sequences, and compared the two distributions of F_ST _values. We excluded all SNPs on the X chromosome, which tend to have higher F_ST _values because of its lower effective population size [26]. We find that F_ST _values in ANC sequences are higher than those for nonaccelerated CNC sequences, but at marginal statistical significance (Mann-Whitney U-test, *P *= 0.0504; Figure 4b). The signal of higher F_ST _values in ANC sequence SNPs becomes significant if we then exclude the SNPs in retroposed genes, pseudogenes, segmental duplications, or CNVs (Mann-Whitney U-test, *P *= 0.0363). SNPs from the studies by Pollard [18] and Prabhakar [19] and their colleagues do not demonstrate a skew in F_ST _values to any statistically significant degree (Additional data file 2).

### Analysis of accelerated noncoding sequences associated with differential gene expression

To assess the functional impact of nucleotide variation in ANC sequences on phenotypic variation, we looked for associations between SNPs from the phase II HapMap [26,27] within ANC sequences or power CNC sequences and gene expression levels from the 210 unrelated HapMap individuals using recently generated gene expression data [30,31] (see Materials and methods, below). We performed a linear regression between quantitative gene expression values for 14,925 probes and numerically coded genotypes of each SNP within a 10 megabase (Mb) window centered on the midpoint of each transcript probe. The statistical significance was evaluated through the use of 10,000 permutations performed separately for each gene to give adjusted significance thresholds of 0.0001, 0.001, and 0.01 (Table 2). At these thresholds we find three, 58, and 458 SNP to gene expression associations for ANC sequences and 43, 135, and 960 SNP to gene expression associations for power CNC sequences, respectively, across all populations. At the 0.01 threshold 16% of the tested ANC sequences (59/366) contain SNPs that are significantly associated with the expression of a gene, contrasting with only 3% of the tested power CNC sequences (165/5968; Table 2). This means that a SNP within an ANC sequence is seven times more likely to be associated with variation in gene expression levels than is a SNP within a power CNC sequence, and that nucleotide variation within ANC sequences is five times more likely to be associated with gene expression levels than variation in a power CNC sequence. At the most stringent threshold three genes are associated with ANC sequences: *C13orf7*, which is of unknown function; *SLC35B3*, a probable sugar transporter; and *RBPSUH *(Recombining Binding Protein SUppressor of Hairless), which is a J kappa-recombination signal-binding protein.

**Table 2:** Summary of SNPs within ANC sequences and power CNC sequences associated to differential gene expression

Population	Sequence	Number of tested ANC/CNC sequences	Number of SNPs	Number of probes tested	Number of associations	Number of significant ANC/CNC sequence to gene associations			Number of significant ANC/CNC sequences of those tested		
						
						0.01	0.001	0.0001	0.01	0.001	0.0001
CEU	ANC	387	555	8,673	23,330	77	9	0	59 (15%)	9 (2%)	0 (0%)
	Power CNC	6,232	8,388	14,906	350,309	181	36	18	149 (2%)	33 (1%)	17 (0%)
CHB	ANC	356	499	8,092	21,291	83	13	0	56 (16%)	11 (3%)	0 (0%)
	Power CNC	5,737	7,579	14,893	317,518	202	41	15	159 (3%)	39 (1%)	15 (0%)
CHB and JPT	ANC	342	466	7,919	20,163	109	11	1	59 (17%)	9 (3%)	1 (0%)
	Power CNC	5,474	7,162	14,852	301,636	203	12	1	149 (3%)	12 (0	1 (0%)
JPT	ANC	355	490	8,197	21,166	88	12	0	59 (17%)	11 (3%)	0 (0%)
	Power CNC	5,674	7,531	14,852	315,476	241	48	20	194 (3%)	42 (1%)	19 (0%)
YRI	ANC	391	583	9,118	24,310	113	15	2	64 (16%)	15 (4%)	2 (1%)
	Power CNC	6,724	9,218	14,908	381,407	196	32	15	173 (3%)	30 (0%)	14 (0%)

Presented are results for four populations: the Yoruba people from Ibadan Nigeria (YRI), US residents with Northern and Western European ancestry (CEU), Han Chinese from Beijing (CHB), and Japanese from Tokyo (JPT). ANC, accelerated noncoding; CNC, conserved noncoding; SNP, single nucleotide polymorphism.

We further explored the biological properties of the associated genes at the significance threshold of 0.01 by counting the occurrences of each of the Gene Ontology (GO) slim terms associated with these genes. We compared the proportions of genes with and without a GO slim term for ANC sequence associated genes versus those tested with the same counts for power CNC sequences (Fisher's exact test). Genes associated with ANC sequence variation are deficient for the GO slim term 'binding' and enriched for the GO slim term 'physiologic process' relative to power CNC sequences. Overall, this suggests that ANC sequence nucleotide variation affects expression of different types of genes to a greater degree than does nucleotide variation within power CNC sequences (after controlling for the types of genes that were included in the analysis), but that the counts are too small to draw specific conclusions about the nature of the effect.

## Discussion

We have detected 1,356 CNC sequences that have an accelerated substitution rate in the human relative to the chimpanzee lineage (human ANC sequences). Misalignment of paralogous sequences is unlikely to explain the overall signal, and manual curation confirms that this only potentially occurs in fewer than 3% of cases. The lower quality of the other two genomes has minimal effect on the human ANC sequence analysis, because for a substitution to be classified as human specific both the chimpanzee and the macaque sequences must have the same nucleotide and differ from the human nucleotide. We therefore expect this test to be conservative because many chimpanzee-specific substitutions could be sequencing errors, leading to an overestimate of these. The comparison of the human substitution rate in control regions 10 kb or 500 kb from power CNC sequences or the expected human synonymous substitution rate (Ks) with that of the ANC sequences suggests that 15% to 19% of the ANC sequences have not simply diverged from the sequence of the common ancestor because of loss of constraint, but that the rate of divergence has increased twofold to fourfold above that expected under neutrality, indicating that they have undergone positive selection.

An interesting possibility is that some ANC sequences are degenerate regulatory elements associated with subfunctionalized duplicate genes, as described in the duplication-degeneration-complementation model [13], or elements that have decayed in function in a similar way to pseudogenes. We found an enrichment of the ANC sequences within the most recent segmental duplications (<2% divergence) relative to both power CNC sequences and nonaccelerated CNC sequences. The general enrichment in segmental duplications is not surprising, because it has been observed that sequence divergence is elevated in duplicated sequences [32,33]. The most recent segmental duplications in the human genome occurred after the human-chimpanzee split, and differential evolution between these copies would explain the human-specific acceleration caused by loss of selective constraint due to redundancy or positive selection due to gain of a new function. The DAF analysis suggests that many newly derived alleles within ANC sequences are undergoing positive selection, there are unfortunately insufficient genotyped SNPs to test those only within segmental duplications.

If the signal of ANC sequences were due to misalignments, then we would have observed an excess of ANC sequences in older and more divergent segmental duplications. We therefore conclude that the recent change in selective forces of some ANC sequences may be a result of duplication.

The overlap of ANC sequences with elements such as retroposed genes and pseudogenes is not surprising because these elements are thought to undergo degradation or change when they are released from the selective constraint placed on active genes. They are, however, more enriched in the power CNC sequences than in the ANC sequences. By parallel analysis we demonstrate that our observations are generally robust to inclusion of ANC sequences in the above elements.

Regions with an excess of SNPs with high DAF relative to the expectations of a neutral equilibrium model are likely to be evolving under positive selection [28]. The DAF spectrum of the ANC sequences exhibits an excess of high-frequency derived alleles relative to the DAF spectrum of all control sets. In addition, the observation of higher population differentiation (higher F_ST _values) in ANC sequence SNPs suggests not only that ANC sequences have contributed to evolutionary change along the human lineage since the time of the human-chimpanzee common ancestor, but also that some have contributed to recent differentiation between human populations. The power CNC sequence set is expected to contain regions that have high substitution rates and also regions with human lineage-specific acceleration that failed to meet the significance threshold for inclusion in the ANC sequence category, or previously fast-evolving regions that have switched selective pressures before the human-chimpanzee split that therefore have similar rates in both human and chimpanzee. This hypothesis is strengthened by the recent study conducted by Pollard and coworkers [18], because 112 out of the 202 HARs overlap the power CNC sequences of the present study. The overlap of 112 HARs with power CNC sequences is not due to low power in our study but mainly due to the fact that our analysis makes the explicit assumption that the human lineage is significantly faster than that of the chimpanzee, which is not the case in the study conducted by Pollard and coworkers. Interestingly, the most significant ANC sequence in our analysis completely overlaps with the most significant element in the Pollard study (HAR1) [34].

We observed that SNPs within ANC sequences are significantly associated with gene expression phenotypes, and the probability that SNP variation within an ANC sequence being associated is fivefold higher than for a power CNC sequence. The pattern of enrichment in gene expression associations provides our strongest evidence that ANC sequences contain functionally evolving sequence that is associated with changes in gene expression. There is a tendency for the derived alleles within ANC sequences to be associated with low gene expression levels, although this is not statistically significant. Because the derived allele is high in frequency in SNPs within ANC sequences this could indicate that low expression could be potentially advantageous for some genes, but this cannot be tested formally with this dataset because of the small sample size.

The presence of ANC sequences in the human genome suggests that the evolution of noncoding DNA contributes substantially to species differentiation. Our analysis relies on the identification of these ANC sequences by initially requiring conservation across multiple vertebrate species, and so it is conservative with respect to the contribution of functional noncoding elements to species differentiation. Previous studies have shown that the proportion of functional noncoding sequences can be large and not necessarily conserved above neutral expectation [3]. When additional genomes become available, increasingly rigorous analyses and detection methodologies can be developed to elucidate the degree of noncoding and regulatory evolution and the birth-and-death process of regulatory elements. Nevertheless, the ANC sequences identified in this study can serve as a baseline for the elucidation of biological processes in noncoding DNA that contribute to species differentiation.

## Materials and methods

### Detection of accelerated noncoding sequences: alignments and calling of accelerated noncoding sequences

CNC sequences were detected using a phylogenetic hidden Markov model (phyloHMM) [35] in the top 5% of the conserved genome (PhastCons conserved elements, 17-way vertebrate MULTIZ alignment), as available at the University of California, Santa Cruz Genome browser [36]. The top 5% represents the minimal selectively constrained genome, as inferred from the Mouse genome analysis [37]. We selected elements of at least 100 bases to increase our power to detect acceleration and intersected those elements with Ensembl gene predictions (v40, August 2006) [38] to obtain the set of elements that did not overlap any part of the processed transcript. CNC sequences with more than four substitutions between human and chimpanzee were aligned among human, chimpanzee, and macaque, and lineage-specific substitutions were inferred assuming parsimony. Alignments of these elements were obtained from a three-way MULTIZ alignment [39] of human finished sequence (hg18), chimpanzee assembly (panTro2), and macaque (draft assembly). The human and chimp genome sequences were aligned with the blastz program [40] with the substitution scores presented in Table 3 and penalizing a gap of length k by 600 + 150 k. The substitution scores for human-rhesus alignments are also summarized in Table 3, and a gap of length k was penalized by 600 + 130 k.

**Table 3:** Substitution score matrices for human-chimp and human-rhesus alignments

Alignment	A	C	G	T
Human-chimp	90	-330	-236	-356
	-330	100	-318	-236
	-236	-318	100	-330
	-356	-236	-330	90
Human-rhesus	87	-226	-129	-255
	-226	100	-212	-129
	-129	-212	100	-226
	-255	-129	-226	87

A three-way alignment of human, chimp, and rhesus was computed using the multiz program [39] and searched for intervals of interest (for example, at least four mismatches) using software written specifically for that purpose.

For the following analysis the human coordinates were mapped from NCBI 36 (hg18) to NCBI 35 (hg17) using the liftOver program [41].

Because we are testing for differences in the relative rates of substitution along the lineages, paralogous alignments of duplicates after the (macaque [chimpanzee, human]) split will not generate a signal because the length of the branches are the same. The only scenario that can generate a false signal is if the duplication occurred before the (macaque [chimpanzee, human]) split, giving rise to copies X and Y, and the alignment is between the chimpanzee and macaque copy X and the human copy Y. This scenario requires that the human copy X has been lost and that the macaque and chimpanzee copies of Y are either not included in the assembly or have also both been lost. The fact that this requires three losses/misses makes the scenario unlikely, and inspection of the data does not suggest that it is occurring.

We applied the χ^2^-based relative rate test [17] to detect sequences that are accelerated in either the human or chimpanzee lineage. Because this method could potentially be affected by small counts of substitutions, we applied the Yates' correction for continuity, which is conservative in estimating the *P *value of the test. We then selected the threshold that had the lowest FDR in the range of *P *values between 0.05 and 0.15. This threshold was *P *= 0.08, with estimated FDR of 75%; we therefore subsequently analyzed all human ANC sequences that have *P *≤ 0.08. Note that the Yate's correction generally over-corrects, and so our FDR is likely to be an overestimate.

As a control for our ability to detect human accelerated regions, we compared the relative enrichment of our ANC sequences and power CNC sequences in those detected as accelerated in humans using alternative methods [18,19]. Although the tests differ in their approaches (ours, for example, conditions on human lineage acceleration versus the chimpanzee lineage only), we find a sixfold enrichment of previously detected accelerated regions (HARs and accelerated CNSs) in our ANC sequence set relative to the power CNC sequences control set.

Because of the lower quality of the chimpanzee and macaque genome sequences relative to the human genome sequence, we only considered sequences accelerated in the human lineage. As a control, we also performed alignments of human-chimpanzee-macaque at coordinates 10 and 500 kb away from the initial CNC sequence coordinates to use as controls for the neutral substitution rate.

### Segmental duplications

A set of genomic coordinates corresponding to segmental duplications, defined elsewhere [21,22], were used as points of reference in the genome. Accelerated, nonaccelerated, and power CNC sequences were then mapped to those segmental duplications, and the abundance of ANC sequences was compared with the observed abundance of nonaccelerated or power CNC sequences in segmental duplications as well as the estimated coverage of the genome by segmental duplications (5% to 6%). CNV genomic coordinates were obtained from the Database of Genomic Variants in Toronto [23].

### Pseudogenes and retroposed genes

Genomic coordinates for retroposed genes and two set of pseudogenes (Yale and Vega annotations available at the University of California, Santa Cruz Genome browser [36]) were used. Accelerated, nonaccelerated, and power CNC sequences were then mapped to those coordinates, and an overlap was defined whenever at least a single base was common between the two sets of features under comparison.

### Single nucleotide polymorphisms and F_ST_values

SNPs from phase I and phase II from release 19 of the HapMap project [26,27] were mapped from NCBI 34 (hg16) to NCBI 35 (hg17) using the liftOver program [41]. SNPs that did not map to hg17 were ignored and derived alleles were inferred based on the chimpanzee alignment to the hg17 version of the human genome. For those SNPs that did not have a reliable chimpanzee alignment, the alignment to the rhesus macaque was used. Inference of the derived allele was based on parsimony, and the common allelic state between the human and the chimpanzee (or macaque in few cases) was considered the ancestral allele. The DAF was estimated and DAF spectra were compared using the nonparametric Mann-Whitney U-test. One potential caveat of this analysis is that, because we required the reference human sequence to be quite divergent from the chimpanzee, we have selected a large number of CNC sequences with an excess of derived alleles by chance, which specifically enriches for SNPs with high DAFs. We find this unlikely because only 4.2% of the fixed differences (281/6,660) that produced the signal of acceleration can be explained by the derived alleles of HapMap SNPs in the reference sequence, and this can only increase to approximately 8% if ungenotyped SNPs are accounted for. Therefore, the bulk of the signal for acceleration was independent of the DAFs of the SNPs within the ANC sequences. The SNP ascertainment does not affect the analysis because we are using both phase I and II SNPs of the HapMap, which together provide a relatively unbiased view of SNP density and allele frequencies. In addition, any potential bias toward genic regions would not create a bias in our analysis because all of the frequency spectra we compare are independent of genes.

The phase II HapMap is estimated to contain more than half of the common SNPs in the tested Yoruban (YRI) Hap Map population, as has been estimated by the resequenced ENCODE regions [26]. Therefore, the contribution of SNPs to divergence is not expected to be more than 8%. This, together with the comparison with the accelerated sequences at 10 kb and 500 kb, suggests that small confounding effects of divergence and DAF spectrum are not the reason for our signal. F_ST _values for each SNP in ANC sequences and nonaccelerated CNC sequences were calculated according to the method proposed by Weir and Cockerham [29]. Distributions of F_ST _values were compared using the Mann-Whitney U-test excluding the X chromosome SNPs. This comparison of distributions was repeated with power CNC sequences, and ANC sequences, excluding any SNPs in segmental duplications, CNV, retroposed genes, or pseudogenes.

### Gene expression associations

We used gene expression data on 47,294 transcripts in lymphoblastoid cell lines of all 210 HapMap [26] unrelated individuals from the four populations, in four technical replicates. The gene expression values of 47,294 transcripts interrogated using the array were then normalized and averages taken for each probe across replicates. We downloaded the HapMap [26,27] genotypes (release 21) for each population of all of the phase II SNPs (with a minor allele frequency >5%) within ANC sequences and power CNC sequences. A linear regression was then performed (separately within each population) between quantitative gene expression values for 14,925 probes (a subset chosen on the basis of sufficient measurable expression levels and variability) and numerically coded genotypes (0, 1, 2) of each SNP within a 10 Mb window centered on the midpoint of each transcript probe. The statistical significance was evaluated through the use of 10,000 permutations performed separately for each gene. In each permutation of a single gene, the most significant *P *value was retained, and so that there were 10,000 *P *values for each gene. From these distributions, for each gene, we determined significance thresholds of 0.0001, 0.001, and 0.01. For each gene tested for association with SNPs in ANC sequences or power CNC sequences, the GO slim terms were tabulated in a nonredundant list (multiple transcripts were removed). For each GO slim term the counts of genes with and without the GO slim term in significantly associated genes (at threshold 0.01) and the total genes tested were compared using 2 × 2 contingency tables tested by the Fisher's exact test for genes associated with SNPs in accelerated and the power CNC sequences.

## Additional data files

The following additional data files are available with the online version of this paper. Additional data file 1 is a table listing the coordinates for ANC sequences, highlighting those manually checked and overlapping other elements. Additional data file 2 is a figure of the patterns and levels of nucleotide variation in ANC sequences compared with the alternatively defined fast-evolving CNC sequences.

## Supplementary Material

Additional data file 1 Provided is a table listing the coordinates for ANC sequences, highlighting those manually checked and overlapping other elements. Click here for file

Additional data file 2 Provided is a figure of the patterns and levels of nucleotide variation in ANC sequences compared to the alternatively defined fast evolving CNC sequences. Click here for file
