# Supplementary material for: Fast-evolving noncoding sequences in the human genome
Source: Genome Biol. 2007 Jun 19;8(6):R118. doi: 10.1186/gb-2007-8-6-r118 (PMC2394770; doi:10.1186/gb-2007-8-6-r118)

Figure S1 - **Patterns and levels of nucleotide variation in ANCs, comparison to alternatively defined fast evolving CNCs.**

(A) The comparative DAF spectra for phase II HapMap SNPs in non-accelerated CNCs (n = 48,811), ANCs (n = 682), ANCs outside of SDs, CNVs, retroposed genes or pseudogenes (n = 610), in the two controls (n = 28,408 and n = 28,722) in the *power CNCs* (n = 10,882), in the HARs from Pollard et al. (n = 84), and in the accelerated CNSs of Prabhakar et al. (n = 328) in the 60 individuals of the Yoruban (YRI) population.

(B) The comparative distributions of F_{ST} values for all phase II HapMap SNPs in
ANCs (n = 688,) ANCs outside of SDs, CNVs, retroposed genes or pseudogenes (n = 620), *power CNCs* (n = 11,267), non-accelerated CNCs (n = 52,210), HARs (n = 82) and accelerated CNSs (n = 340).

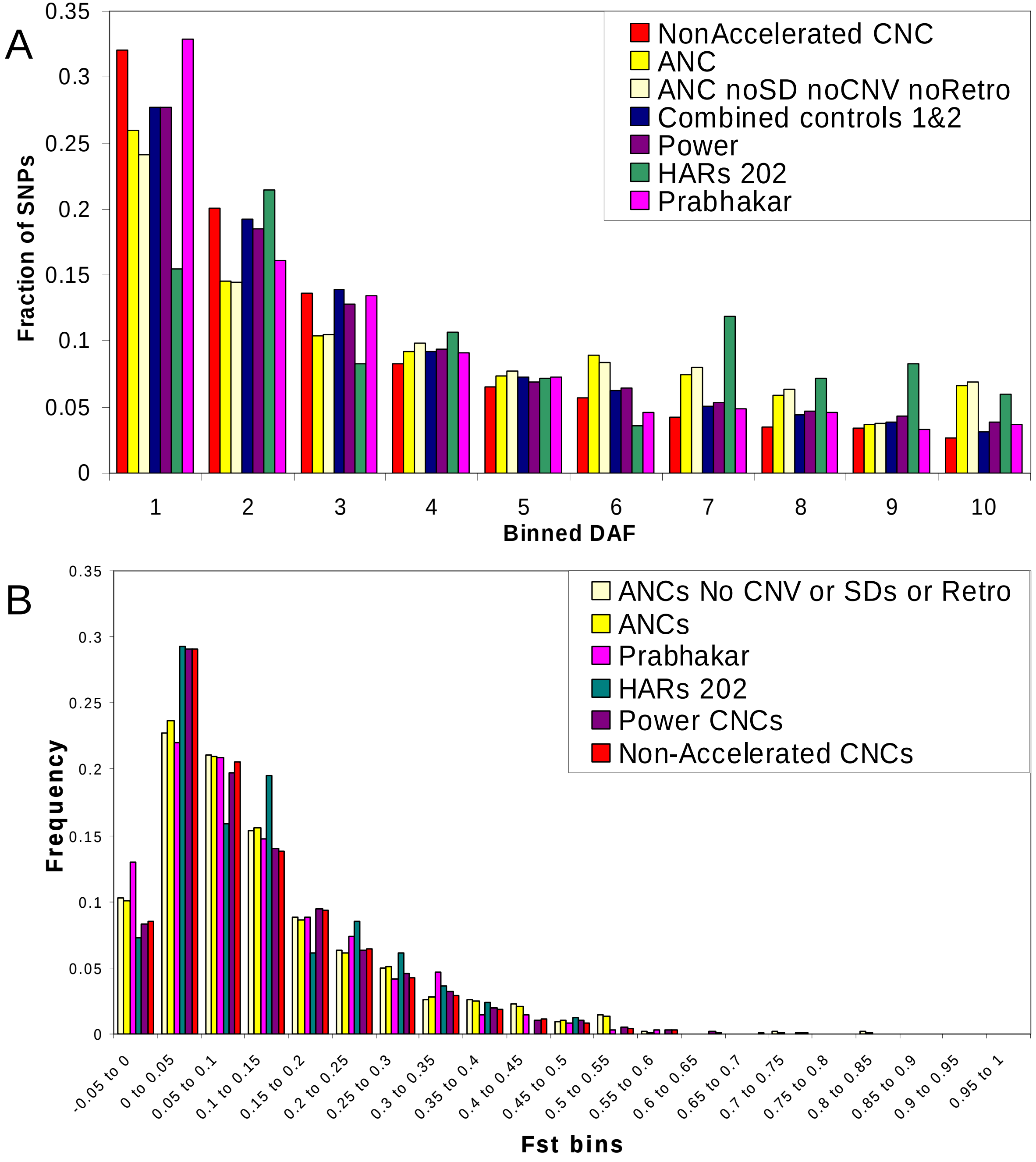

Supplement: Additional data file 2 — Provided is a figure of the patterns and levels of nucleotide variation in ANC sequences compared to the alternatively defined fast evolving CNC sequences. [file gb-2007-8-6-r118-S2.pdf]
